# Intraperitoneal Administration of a Novel TAT-BDNF Peptide Ameliorates Cognitive Impairments via Modulating Multiple Pathways in Two Alzheimer’s Rodent Models

**DOI:** 10.1038/srep15032

**Published:** 2015-10-14

**Authors:** Yuanyuan Wu, Xiaobin Luo, Xinhua Liu, Deyi Liu, Xiong Wang, Ziyuan Guo, Lingqiang Zhu, Qing Tian, Xifei Yang, Jian-Zhi Wang

**Affiliations:** 1 Department of Pathophysiology, School of Basic Medicine and the Collaborative Innovation Center for Brain Science, Key Laboratory of Ministry of Education of China for Neurological Disorders, Tongji Medical College, Huazhong University of Science and Technology, Wuhan 430030, China; 2 Department of Anesthesiology, Wuhan Children Hospital, Wuhan 430030 China; 3 Key Laboratory of Modern Toxicology of Shenzhen, Shenzhen Center for Disease Control and Prevention, Shenzhen, 518055, China; 4 Shenzhen/Guangzhou Kai-Tuo Biotech, Guangzhou, 510800, China; 5 Co-innovation Center of Neuroregeneration, Nantong 226001, China

## Abstract

Although Alzheimer’s disease (AD) has been reported for more than 100 years, there is still a lack of effective cures for this devastating disorder. Among the various obstacles that hold back drug development, the blood-brain barrier (BBB) is one of them. Here, we constructed a novel fusion peptide by linking the active domain of brain-derived neurotrophic factor (BDNF) with an HIV-encoded transactivator of transcription (TAT) that has a strong membrane-penetrating property. After intraperitoneal injection, the eGFP-TAT could be robustly detected in different brain regions. By using scopolamine-induced rats and APPswe mice representing AD-like cholinergic deficits and amyloidosis, respectively, we found that intraperitoneal administration of the peptide significantly improved spatial memory with activation of the TrkB/ERK1/2/Akt pathway and restoration of several memory-associated proteins in both models. Administration of the peptide also modulated β-amyloid and tau pathologies in APPswe mice, and it increased the amount of M receptor with modulation of acetylcholinesterase in scopolamine-induced rats. We conclude that intraperitoneal administration of our TAT-BDNF peptide could efficiently target multiple molecular pathways in the brain and improve the cognitive functions in AD-like rodent models.

Alzheimer’s disease (AD) is the most common cause of senile dementia, and it has severe impacts on a patient’s overall health status, as well as their social well-being^1^. The principle pathological features of AD involve formation of intraneuronal neurofibrillary tangles (NFTs), which are mainly composed of the hyperphosphorylated microtubule-associated protein tau, and senile plaques (SP) resulting from an extracellular deposition of β-amyloid (Aβ). The major clinical manifestation of AD is progressive memory loss^2^,^3^. Several lines of evidence show that cholinergic dysfunction is an early event of AD^4^,^5^,^6^.

Presently, the drugs approved by the United States Food and Drug Administration (FDA) for the clinical treatment of AD generally fall into two categories, i.e., acetylcholinesterase (AChE) inhibitors, such as tacrine, donepexil, and rivastigmine, and N-methyl-D-aspartate (NMDA) receptor antagonists, such as memantine. These AD drugs are known to be effective only in mild and moderate AD patients by improving clinical symptoms^7^,^8^. Thus, there is an urgent need for improved therapies that can cure AD or delay AD progression.

One potentially promising target for novel AD drug development is brain-derived neurotrophic factor (BDNF). BDNF is known to promote axonal and dendritic growth and neural repair in response to damage. BDNF can enhance survival of various types of neurons via improving intracellular antioxidant enzyme activities^9^,^10^, promote long-term potentiation, increase synaptic plasticity, and stimulate release of acetylcholine into the synaptic cleft *in vitro*^11^,^12^. The neuroprotective property of BDNF has also been demonstrated in AD animal models^13^. These data suggest that targeting BDNF is promising for AD. However, one major obstacle for the clinical application of BDNF is that this molecule cannot cross blood-brain barrier, mainly due to its large molecular weight.

After analyzing the crystal structure of BNDF, O’Leary and Hughes found that a dimeric peptide mimetic of BDNF loop 2 could bind to the tropomyosine-related kinase B (TrkB) receptor with high affinity and play a protective role *in vitro*^14^. It has not been elucidated whether this active BDNF peptide is also neuroprotective *in vivo*. Frankel and Pabo reported that a transactivator of transcription (TAT) encoded by the human immunodeficiency virus-1 (HIV-1) has a strong membrane-penetrating property^15^,^16^. The transduction domain of TAT contains 11 basic amino acids (YGRKKRRQRRR) that determine the penetrating ability of the molecule^17^.

In the present study, we constructed a fusion peptide by linking the core functional domain of BDNF, referring to a previous study^14^, with a TAT transduction domain (Supplementary Fig. 1) and investigated the neuroprotective effects of the TAT-BDNF peptide in two AD-like animal models. We found that the TAT could efficiently carry eGFP across the brain-blood barrier (BBB) after an intraperitoneal injection. Intraperitoneal administration of the TAT-BDNF fusion peptide remarkably improved spatial memory. The mechanisms involve activation of the TrkB receptor-associated signaling pathway, including activation of the extracellular signal-regulated kinase 1/2 (Erk1/2), phosphatidylinositol 3-kinase/protein kinase B (PI3K/Akt), upregulation of CREB and Arc, as well as some synapse-associated proteins. The peptide also ameliorated cholinergic dysfunction induced by scopolamine and the neuropathologies caused by amyloid. We propose that peripheral administration of the novel TAT-BDNF fusion peptide may represent a new direction for drug development for AD.

## Results

### The eGFP-TAT peptide can penetrate the BBB into the brain after intraperitoneal injection

To test whether TAT can effectively across the BBB, we injected eGFP-TAT intraperitoneally into rats. After 4 h, a robust eGFP signal was observed in the hippocampus, hypothalamus, and amygdala of the brain. By co-staining with Hoechst, we found that the rate of eGFP-positive cells was approximately 70% in brain areas with a rich blood supply, such as the hippocampal CA3 region, hypothalamus, and amygdala, and approximately 60% in the entorhinal cortex, and hippocampal CA1 and CA2 regions (Supplementary Fig. 2A,B). These data suggest that TAT can efficiently transport macromolecules (eGFP) across the BBB.

### Intraperitoneal administration of the TAT-BDNF fusion peptide improves memory deficits in APPswe mice and scopolamine-induced rats

**Figure 1:**
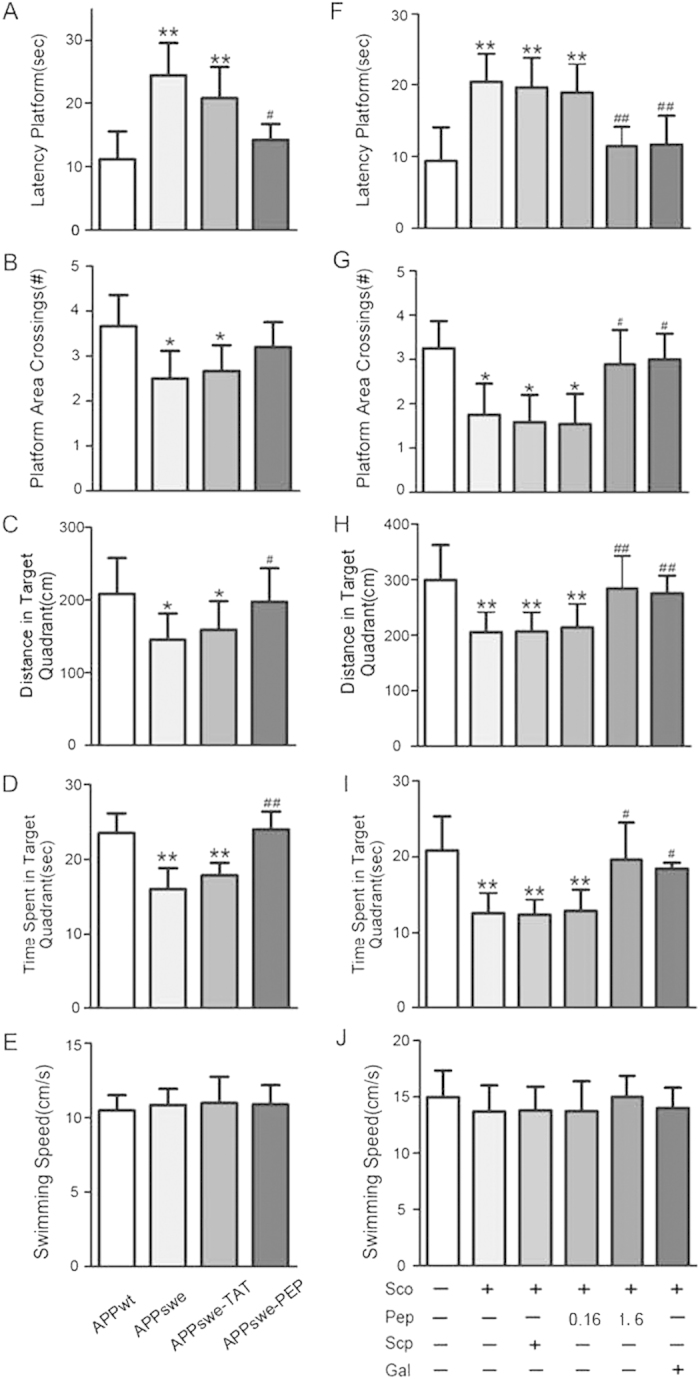
Intraperitoneal administration of the TAT-BDNF fusion peptide improves memory deficits in APPswe mice and scopolamine-induced rats. APPswe mice (12 month old) (*n *= 10) (**A**–**E**) or SD rats (*n *= 10) (**F**–**J**) were injected respectively via intraperitoneal with saline (vehicle control), or TAT (vector control), or TAT-BDNF (PEP, 0.16 μg/day and 1.6 μg/day for one week in rats, 2.4 μg/day for one month in mice). The spatial learning and memory were measured by water maze test. The age-matched wild type littermates were used as naïve control of APPswe. To produce AD-like cholinergic dysfunction model, scopolamine (Sco, 1 mg/kg) was injected through peritoneal 0.5 h before the behavior test. The scrambled TAT-BDNF (Scp, 1.6 μg/day) and galantamine (Gal, 3.0 mg/kg) were used respectively as negative and positive controls. *p < 0.05, **p < 0.01 *vs* Con (APPwt or normal rats), #*p *< 0.05, ##*p *< 0.01 *vs* APPswe group.

APPswe and wild type (APPwt) mice at 12 months of age were given a daily intraperitoneal injection with the TAT-BDNF peptide (PEP, 2.4 μg/day), TAT (vector control), or saline (vehicle control) for one month, and then spatial learning and memory were tested using the Morris water maze test. Compared with APPwt mice, the learning and memory abilities were significantly impaired in APPswe mice (Fig. 1A–E). Administration of the TAT-BDNF peptide reduced the latency to find the platform (Fig. 1A) and increased the time and path length in the target quadrant (Fig. 1B–D), suggesting that the TAT-BDNF peptide could improve learning and memory. Treatment with TAT alone did not alter the cognitive behavior in APPswe mice (Fig. 1A–D). We also recorded swimming speed during the test, and there was no significant difference among the groups (Fig. 1E), suggesting that the changes in learning and memory were not caused by locomotor differences.

As reported previously, an injection of scopolamine (Sco) caused learning and memory deficits, while pre-injection of the rats with the TAT-BDNF peptide (1.6 μg/day) for one week prevented the scopolamine-induced impairment of learning and memory (Fig. 1F–I). The effects of the peptide were similar to galantamine (Gal), a cholinergic stimulator used as a positive control (Fig. 1F–I). No significant difference in swimming speed was detected among the groups (Fig. 1J).

### Intraperitoneal administration of the TAT-BDNF fusion peptide stimulates the TrkB, Erk, and Akt pathways in the hippocampi of APPswe mice and scopolamine-infused rats

**Figure 2:**
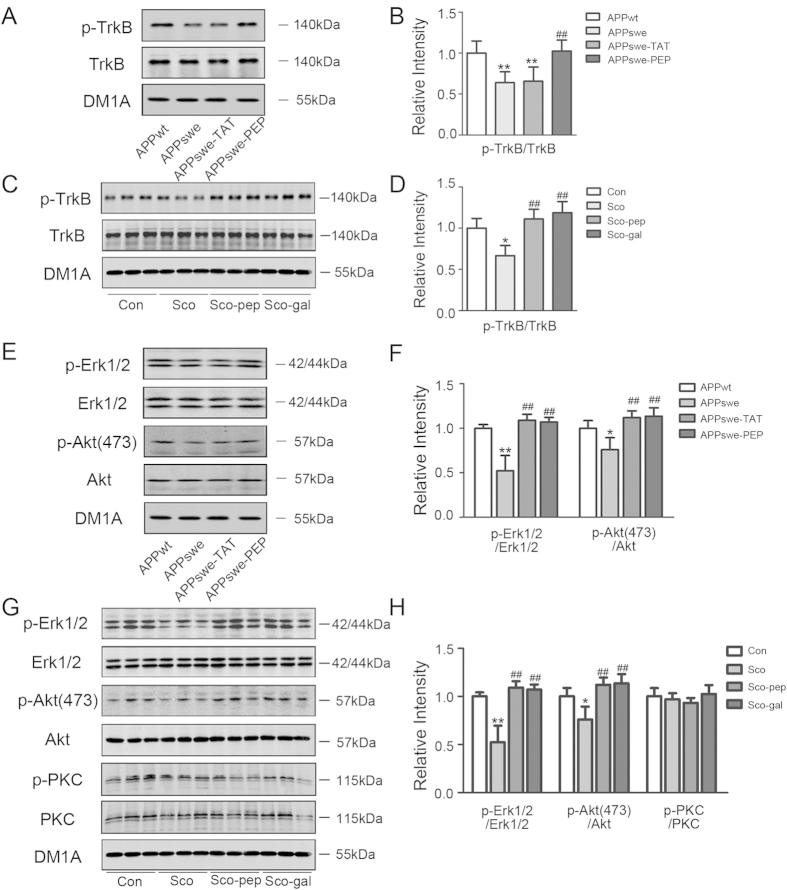
Intraperitoneal administration of the TAT-BDNF fusion peptide stimulates TrkB/Erk/Akt pathway in hippocampus of APPswe mice and scopolamine-infused rats. The mice (*n *= 3) (**A**,**B**,**E**,**F**) and rats (*n *= 5) (**C**,**D**,**G**,**H**) were treated as described in Fig. 1. After behavior test, the animals were sacrificed and the hippocampal extracts were prepared for Western blotting and quantitative analyses of TrkB (**A**–**D**) and Erk/Akt (E-H). Levels of the phosphorylated TrkB, Akt(473), Erk1/2, and PKC were normalized against total levels of the correlated kinases. The total levels of the kinases were normalized against tubulin probed by DM1A. *p < 0.05, **p < 0.01 *vs* Con (APPwt or normal rats), #*p *< 0.05, ##*p *< 0.01 *vs* APPswe group or Sco group.

Using western blotting, we observed that the level of phosphorylated TrkB (p-TrkB) was significantly lower in APPswe than APPwt mice and that administration of the TAT-BDNF peptide restored p-TrkB to the normal level in APPswe mice (Fig. 2A,B). Compared with the vehicle control rats, p-TrkB was significantly decreased in Sco-infused rats, and the TAT-BDNF peptide restored the p-TrkB level, as it did by galantamine (Fig. 2C,D). No significant difference in total TrkB protein was observed among the different groups (Fig. 2A–D). These data demonstrate that the TAT-BDNF peptide maintains the TrkB receptor ligand activity and that it can cross the brain-blood barrier to activate TrkB receptors in the brain.

The downstream signaling pathways activated by the TrkB receptor include the MAPK/Erk1/2, PI3K/Akt, and PLCγ pathways. Therefore, we measured the expression and phosphorylation of Erk1/2, Akt (Ser473), and PKC (downstream target of the PLCγ pathway) in the hippocampi of the APP mice and Sco-infused rats using western blotting. The results showed that the levels of p-Akt (active form) and p-Erk1/2 were significantly decreased in APPswe mice compared with APPwt mice and that administration of the TAT-BDNF peptide restored the levels of p-Akt and p-Erk1/2 in APPswe mice (Fig. 2E,F). In Sco-treated rats, the levels of p-Erk1/2 and p-Akt were significantly decreased, and administration of the TAT-BDNF peptide or galantamine restored the levels of p-Erk1/2 and p-Akt (Fig. 2G,H). In contrast, the expression levels of p-PKC and total PKC were not significantly changed among the groups (Fig. 2G,H). These data suggest that activation of Erk1/2 and Akt, but not PKC, is involved in the TAT-BDNF peptide-mediated improvement in learning and memory abilities in the animals.

### Intraperitoneal administration of the TAT-BDNF fusion peptide up-regulates Arc or CREB in the hippocampi of APPswe mice and Sco-infused rats

**Figure 3:**
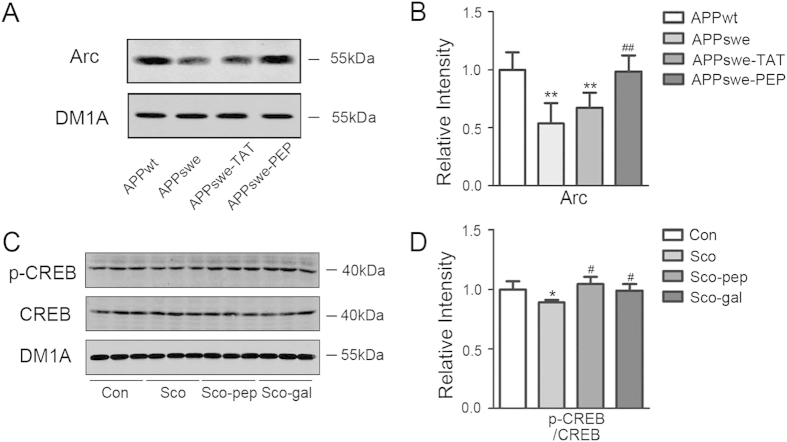
Intraperitoneal administration of the TAT-BDNF fusion peptide up-regulates Arc or pCREB in hippocampus of APPswe mice and Sco-infused rats. The mice and rats were treated as described in Fig. 1. After behavior test, the animals were sacrificed and the hippocampal extracts were prepared for Western blotting and quantitative analyses of Arc (*n *= 3) (**A**,**B**) and CREB (*n *= 5) (**C**,**D**). The level of p-CREB was normalized against total CREB. The total levels of CREB and Arc were normalized against tubulin probed by DM1A. *p < 0.05, **p < 0.01 *vs* Con (APPwt or normal rats), #*p *< 0.05, ##*p *< 0.01 *vs* APPswe group or Sco group.

Arc is an immediate early gene related to learning and memory. In APPswe mice, the expression of Arc was significantly decreased compared to APPwt mice, and administration of the TAT-BDNF peptide restored the level of Arc in APPswe mice (Fig. 3A,B).

The cAMP response element-binding protein (CREB) is an important transcription factor for the regulation of advanced functions in the brain, including learning and memory. The activity of CREB is regulated by many signaling molecules, including Erk1/2 in the MAPK family, and phosphorylation at Ser133 site is necessary for transcription initiation by CREB. Therefore, we examined the expression levels of total and phosphorylated CREB (Ser133). We found that scopolamine decreased the level of p-CREB and that administration of the TAT-BDNF peptide or galantamine restored p-CREB to the normal control level (Fig. 3C,D).

### Intraperitoneal administration of the TAT-BDNF fusion peptide remodels synaptic plasticity in the hippocampi of APPswe mice and Sco-infused rats

**Figure 4:**
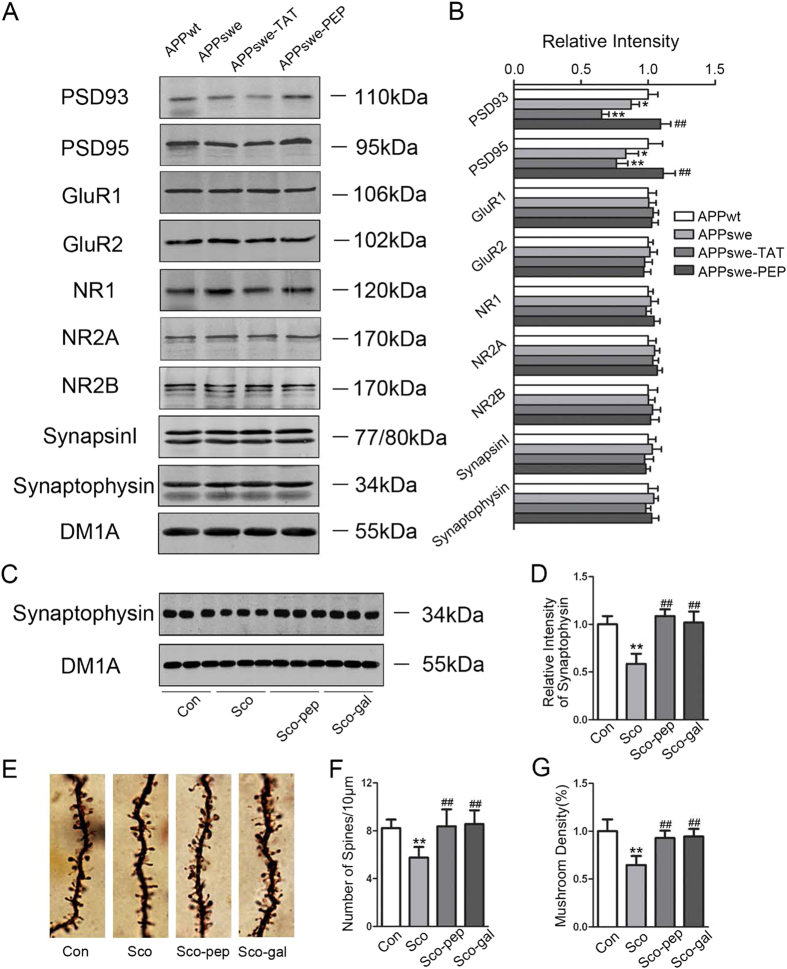
Intraperitoneal administration of the TAT-BDNF fusion *p*eptide remodels syna*p*tic plasticity in hippocampus of APPswe mice and Sco-infused rats. The mice and rats were treated as described in Fig. 1. The levels of PSD93, PSD95, GluR1, GluR2, NR1, NR2A, NR2B, Synapsin I, and Synaptophysin in hippocampal P2 fraction of mice (*n *= 3) (**A**,**B**) or rats (*n *= 5) (**C**,**D**) were measured by Western blotting. The dendrite morphology was shown by Golgi staining (**E**), and spi*n*e density (**F**) and the mushroom-shaped spines (**G**) were analyzed by Image Pro-Plus software (*n *= 30). DM1A against tubulin was used as a loading control. *p < 0.05, **p < 0.01 *vs* Con (APPwt or normal rats), ##*p *< 0.01 *vs* APPswe group or Sco group.

To further investigate the effect of the TAT-BDNF peptide on the expression of synapse-associated proteins, we isolated synaptosomes from the hippocampi of APPswe mice and Sco-infused rats and measured the levels of PSD93, PSD95, GluR1, GluR2, NR1, NR2A, NR2B, synapsin I, and synaptophysin by western blotting. We found that only the levels of PSD93 and PSD95 were significantly decreased in 12-month-old APPswe mice and that administration of the TAT-BDNF peptide restored the levels of PSD93 and PSD95 (Fig. 4A,B). In Sco-induced rats, the level of synaptophysin was decreased, and administration of the peptide restored the level in the hippocampus (Fig. 4C,D, and Supplementary Fig. 3).

Dendritic spines are small functional protrusions found along the neuronal dendritic branch that receive signals from other cells and relay those signals to the cell body. Dendritic filopodia are believed to be the precursor of dendritic spines, whereas mushroom-shaped dendritic spines are functionally mature and can enhance synaptic functions. Alterations in morphology and the number of dendritic spines are some of the most important aspects of synaptic plasticity in the CNS and are closely associated with the strength of learning and memory. To analyze neuronal morphology, we used Golgi staining and analyzed the number of dendritic spines and the percentage of mushroom-shaped spines in hippocampal neurons with high magnification under a microscope. Compared to the normal control group, the number of dendritic spines and the percentage of mushroom-shaped spines were significantly reduced in the Sco group. In contrast, the number of dendritic spines and the percentage of mushroom-shaped spines were significantly increased in rats treated with the TAT-BDNF peptide or galantamine (Fig. 4E–G). These data suggest that the TAT-BDNF peptide can remodel synaptic plasticity.

### Intraperitoneal administration of the TAT-BDNF fusion peptide attenuates Aβ and tau pathologies by inhibiting GSK-3β in the hippocampi of APPswe mice

**Figure 5:**
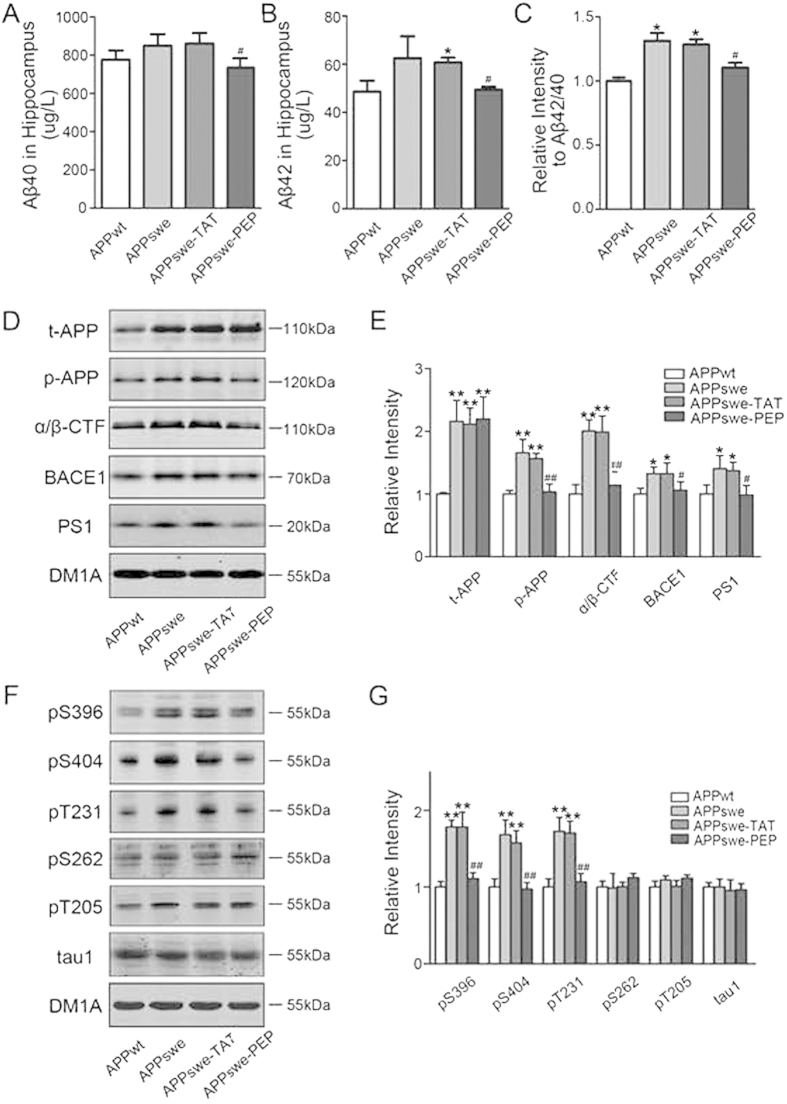
Intraperitoneal administration of the TAT-BDNF fusion pe*p*tide attenuates Aβ and tau pathologies in hippocam*p*i of APPswe mice. The mice were treated as described in Fig. 1. The levels of Aβ40, Aβ42 and the ratio of Aβ42/Aβ40 in hippocampus extract were measured by ELISA (*n *= 3) (**A**,**B**,**C**). The total APP (t-APP), phosphorylated APP at Thr668 (p-APP), and α/β-CTF, BACE1 and PS1 (*n *= 3) (**D**,**E**), the phosphorylated tau (*n *= 3) (**F**,**G**) were measured by Western blotting. DM1A to tubulin was used as a loading control. *p < 0.05, **p < 0.01 *vs* APPwt group, #*p *< 0.05, ##*p *< 0.01 *vs* APPswe group.

Using ELISA, we found that administration of the TAT-BDNF peptide significantly reduced the levels of Aβ42, Aβ40 and the ratio of Aβ42/Aβ40 (Fig. 5A–C), and using immunohistochemistry, we found that administration of the TAT-BDNF fusion peptide significantly reduced Aβ levels (Supplementary Fig. 4).

Phosphorylation of APP at Thr668 (p-APP) and upregulation of β- (BACE1) and/or γ-secretase (PS1) contribute to Aβ overproduction^18^,^19^. We found that the levels of total APP (t-APP), p-APP, α/β-CTF, BACE1, and PS1 were significantly increased in APPswe mice, while administration of the fusion protein reduced the levels of the aforementioned proteins except t-APP (Fig. 5D,E). Additionally, increased tau phosphorylation at Ser396, Ser404, and Thr231 was detected in APPswe mice, and administration of the fusion protein remarkably reduced the phosphorylation of tau (Fig. 5F,G).

**Figure 6:**
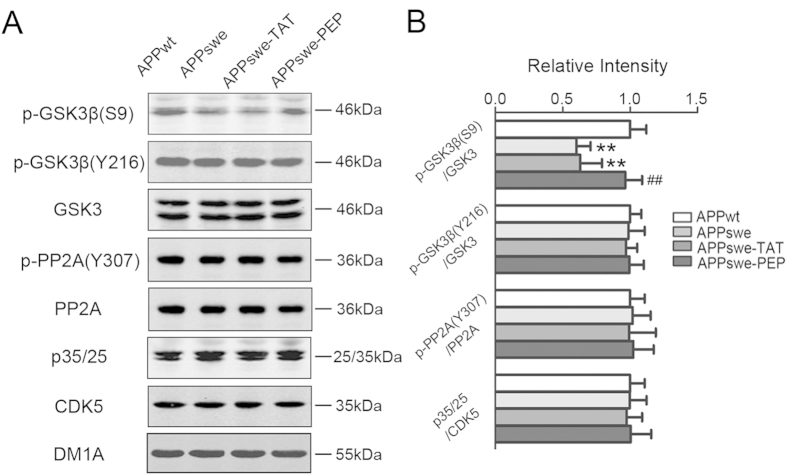
Intraperitoneal administration of the TAT-BDNF fusion peptide modulates the relative activity of GSK3β in hi*p*pocampus of APPswe mice. The rats were treated as described in Fig. 1. The levels of total GSK-3β, p-GSK3β (S9) (inactive form), and p-GSK3β(Y216) (activated form); total PP2A and p-PP2A(Y307) (activated form); CDK5 and its activator P35/25 were measured by Western blotting (*n *= 3) (**A**) and quantitative analyses (**B**). The phosphorylation level of kinases and PP2A was normalized against total level, and the total level of the kinases and PP2A was normalized to tubulin probed by DM1A. **p < 0.01 *vs* APPwt group, ##*p *< 0.01 *vs* APPswe group.

Tau phosphorylation is regulated by protein kinases and phosphatases, and among them, glycogen synthase kinase-3β (GSK3β), cyclin-dependent kinase-5 (CDK5), and protein phosphatase2A (PP2A) are the most implicated in AD^20^. We found that the inhibitory phosphorylation of GSK3β at Ser9 was significantly reduced in APPswe mice and that administration of the peptide restored the level (Fig. 6A,B). On the other hand, the total level of CDK-5, its co-activators p35/p25 and PP2A, its inhibitory phosphorylation at Tyr307, and the activation-dependent phosphorylation of GSK3β at Tyr216 were not changed in 12-month-old APPswe mice, and they were not affected by the TAT-BDNF peptide (Fig. 6A,B). These data suggest that GSK-3β inhibition contributes to the reduced tau and APP phosphorylation induced by the TAT-BDNF peptide.

### Intraperitoneal administration of the TAT-BDNF fusion peptide modulates the cholinergic metabolism in Sco-infused rats

**Figure 7:**
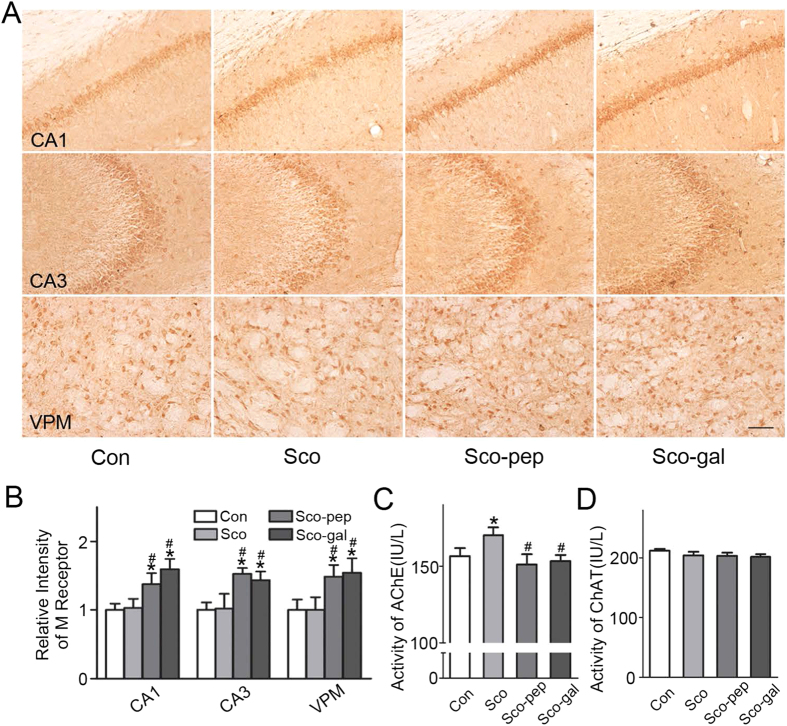
Intraperitoneal administration of the TAT-BDNF fusion peptide modulates the cholinergic metabolisms in Sco-infused rats. The rats were treated as described in Fig. 1. M1 receptor in hippocampal CA1, CA3, and VPM regions was detected by immunohistochemistry (*n *= 3) (**A**) and quantitative analyses (**B**). The activity of AchE and ChAT was measured by ELISA (*n *= 3) (**C**,**D**). *p < 0.05 VS Con group, #*p *< 0.05 *vs* Sco group.

Scopolamine is a muscarinic cholinergic receptor antagonist that disrupts the cholinergic system. To explore whether the TAT-BDNF peptide affected the cholinergic system, we examined the activities of acetylcholinesterase (AChE), choline acetyltransferase (ChAT), and the expression of muscarinic cholinergic receptors. We found that scopolamine did not affect the expression of the M1 receptor in the hippocampus or the ventral posteromedial (VPM) region, while both the TAT-BDNF peptide and galantamine enhanced the expression of the M1 receptor (Fig. 7A,B). The TAT-BDNF peptide and galantamine significantly inhibited the Sco-induced increase in AChE activity measured by ELISA with no effects on ChAT activity (Fig. 7C,D).

### Brain infusion of scopolamine and intraperitoneal injection of the TAT-BDNF fusion peptide do not cause cell death

We also investigated whether the changes in learning and memory were associated with neural death in the hippocampus and cerebral cortex using Nissl staining. No significant differences were detected among the different groups in the hippocampal regions CA1, CA3, DG, or the cerebral cortex (Supplementary Fig. 5), suggesting that the behavioral changes in these rodent AD-like models were not caused by neural loss.

## Discussion

AD is characterized by severe neurological and synaptic degeneration leading to learning and memory deficits. Developing effective AD drugs is a long-standing challenge for AD research. BDNF is a well-established neurotrophic factor that promotes neuronal development, proliferation, differentiation, and repair. While BDNF emerges as a promising target for AD therapeutics^21^,^22^,^23^, the clinical application is largely hindered by its large molecular size, which prevents it from crossing the blood-brain barrier. In addition, the clinical application of BDNF is also hampered by inconvenient drug administration and poor control of drug concentration^9^,^24^,^25^,^26^.

The truncated form of BDNF contains multiple disulfide bonds, which make the peptide structurally more stable^13^. Our results in the present study also show that the BDNF peptide still maintains its bio-activity and neuroprotective functions, such as the ability to bind to the TrkB receptor, after fusion with TAT. The conjugation of the TAT protein transduction domain allows for the transport of macromolecules across the membrane. TAT has become a focus of research since Green *et al.*^27^ and Frankel *et al.*^28^ reported that it can penetrate the plasma membrane. Schwarze *et al.*^29^ fused TAT with 120-kDa galactosidase and intraperitoneally injected the fusion protein into mice. They found that the fusion protein was distributed in the cerebral parenchyma. To date, TAT-mediated fusion proteins, including TAT-GDNF^30^, TAT-Bcl-xL^31^,^32^, and TAT-SOD^14^,^33^ have been used in animal models of local or global brain ischemia. Our current results demonstrated that TAT was capable of carrying eGFP across the blood-brain barrier. When it was fused with the BDNF peptide, the neuroprotective role of the peptide was also shown in two AD-like rodent models after peripheral injection.

Scopolamine induced learning and memory impairments, as reported previously^34^, while administration of the BDNF peptide attenuated the cognitive impairments. Scopolamine is a muscarinic cholinergic receptor antagonist, which can compete with acetylcholine for receptor sites and inhibit normal acetylcholine neurotransmission in the cerebral cortex and hippocampus, thus leading to memory deficits^35^,^36^. Inhibition of acetylcholine neurotransmission increases acetylcholinesterase activity^37^,^38^. We observed that scopolamine increased acetylcholinesterase activity with no effect on the membranous distribution of M1 receptors and the activity of cholineacetyltransferase. Although scopolamine showed no effect on the distribution of M1 receptors, the TAT-BDNF fusion peptide promoted the expression of M1 receptors on the membrane, and that may have contributed to the modulated cholinergic function.

We also found that scopolamine significantly reduced the phosphorylation of TrkB and inhibited its downstream signaling molecules, such as Erk1/2 and Akt. The pathogenic effect of scopolamine is likely attributed to the reduced cerebral blood perfusion and decreased hippocampal volume^15^,^39^,^40^. Scopolamine-induced inhibition of Akt is believed to be one of the most important contributors to scopolamine-induced impairment of learning and memory in AD animals^41^. The TAT-BDNF fusion peptide could also rescue the scopolamine-induced inhibition of the TrkB signaling pathway. It seems that the novel TAT-BDNF peptide could exert its protective functions by targeting both the membranous receptors and some intracellular molecules, which deserves further investigation. These data are in agreement with the concept that BDNF is effective as an AD therapeutic agent, which has been extensively examined using small molecular TrkB agonists, including 7,8-dihydroxyflavone by recent studies^42^,^43^,^44^.

APPswe mice showed memory deficits at 12 months old as reported previously^45^. Therefore, we chose to inject the mice at this age to explore the neuroprotective effects of the new peptide. We found that intraperitoneal administration of the peptide significantly improved the spatial learning and memory abilities in these mice. Given that there was no significant difference in the swimming speed among mice of different groups, we can exclude the possibility that locomotor activity had an effect on learning and memory abilities. We further demonstrated that the TAT-BDNF fusion peptide reduced the Aβ level, likely via the inhibition of BACE1 and PS1, and decreased tau hyperphosphorylation. In addition, the TAT-BDNF fusion peptide increased the expression of PSD93 and PSD95 in the synaptosome and promoted the expression of the immediate early gene Arc. The immediate early gene Arc is a member of a group of genes that were first found to be expressed in response to external stimuli, and arc has been shown to be a mediator that links intracellular biochemical reactions with specific responses to external stimuli^46^,^47^. We found that the TAT-BDNF fusion peptide exhibited its neuroprotective roles via a similar mechanism of TrkB receptor activation and its downstream signaling transduction molecules in both AD-like animal models. In APPswe mice, Akt activation may be a key step for the TAT-BDNF fusion peptide-induced neuroprotection because Akt activation can result in GSK-3β inhibition^48^, which is a key protein kinase in tau hyperphosphorylation at the Ser396, Ser404, and Thr231 sites.

In summary, we have designed a novel fusion peptide composed of the core functional domain of BDNF and the membrane-penetrating TAT. We found that intraperitoneal injection of this peptide could remarkably improve learning and memory in two AD-like rodent models. The mechanisms involve activation of the TrkB/Erk/Akt signaling pathway and attenuation of AD-like cholinergic dysfunction and neural pathologies.

## Materials and Methods

### Chemicals and antibodies

The bicinchoninic acid (BCA) protein detection kit, chemiluminescent substrate kit and phosphocellulose units were from Pierce Chemical Company (Rockford, IL, USA). Alexa Fluor® 568 goat anti-mouse IgG (H+L) was from Molecular Probes (Eugene, OR, USA). The immunohistochemical kit (Histostain-SP) was from ZEMED Company (South San Francisco, CA, USA), and the DAB kit was from ZSGB-Bio. Co., Ltd. (Beijing, China). The Odyssey two-color infrared fluorescence imaging system reagent was from LI-COR Biosciences (USA). Scopolamine Hydrobromide was from Wuhan Yuancheng Tech. Co., Ltd. (Wuhan, China). Galanthamine hydrochloride was from Shanghai Xudong Haipu Pharmaceutical Co., Ltd. (Shanghai, China). The ELISA assay kits for rat acetylcholinesterase, mouse β-amyloid_1–42_ (Aβ42), and mouse β-amyloid_1–40_ (Aβ40) were from Wuhan Biofavor Biotechnology Co., Ltd. (Wuhan, China). The amino acid sequence and structure of the TAT-BDNF peptide are shown in Supplementary Fig. 1, and the peptides were synthesized in the Shanghai Science Peptide Biological Technology Co., LTD. The primary antibodies are listed below (Table 1). The secondary antibodies for western blot analysis were from Amersham Pharmacia Biotech (Little Chalfort, Buckinghamshire, England). pAb, polyclonal antibody; mAb, monoclonal antibody; WB, western blotting; IHC, immunohistochemistry.

### Animals and treatment

The male Sprague-Dawley (SD) rats (Grade SPF, 250–300 g) were supplied by the Experimental Animal Central of Tongji Medical College. The animal experiments were approved by the Animal Care and Use Committee at the Huazhong University of Science and Technology and were performed in compliance with the National Institutes of Health guide for the care and use of laboratory animals. Tg2576 mice express human Swedish mutant APP (APPswe). All animals were housed with accessible food and water ad libitum. All animal experiments were performed according to the “Policies on the Use of Animals and Humans in Neuroscience Research” revised and approved by the Society for Neuroscience in 1995. The animals were kept in cages under a 12:12 light-dark (L/D) cycle with the light on from 7:00 am to 7:00 pm. Housing and hypoxic treatment were done at a stable temperature (23–25 °C) and humidity.

Before the experiments, the animals were allowed to habituate to the laboratory conditions for one week. To produce the rat model with an AD-like cholinergic dysfunction, scopolamine (1 mg/kg) was injected into the peritoneum 0.5 h before water maze training^49^. The rats were randomly divided into six groups: a vehicle control group that was injected with 0.9% NaCl (Con), a scopolamine-induced group (Sco), a negative control group that was injected with a scrambled peptide (Scp), a peptide-treated group that received 0.16 μg or 1.6 μg of the TAT-BDNF fusion peptide, and a positive control group that received the FDA-approved drug galantamine (Gal) (3 mg/kg). APPswe mice at 12 months old that showed memory deficits and AD-like amyloidosis and tau pathologies were used for the study. The injection volume was 1–2 ml/100 g for rats and 0.1–0.2 ml/10 g for mice, and the animals were injected daily for one month before behavioral testing.

### Morris water maze test

The water maze tests were conducted as described previously^50^,^51^. The animals were brought to the site 2 h before the test to allow them to be acclimatized. The test subjects were kept in cages on outer-room shelves to eliminate directional olfactory and auditory cues. The temperatures of the room and water were kept at 25 ± 2 °C. The water in the pool was made opaque with ink to hide the escape platform. The Plexiglas platform was 40 cm high, 10 cm in diameter, and its surface was 2 cm lower than the water surface and was scarred to help the rats to climb on. The diameter and the depth of the water was 150 cm and 60 cm, respectively, and the inner wall was always carefully wiped to eliminate any local cues. A camera was placed 1.5 m above the water surface. The camera was connected to a digital tracking device. An IBM computer with the water-maze software was used to process the tracking information.

The submerged platform (2 cm under the water) was located at a fixed position throughout training. A training session consisted of four trials altogether (one trial per quadrant), with a 20-s interval, lasting for 6 days. During each trial, the rat started from the middle of one of the four quadrants facing the wall of the pool and ended when it climbed onto the platform. The rats were not allowed to search for it for more than 60 s, after which they were guided to the platform. On the seventh day, rats were trained in the absence of the Plexiglas platform, and their escape latency and swimming path were recorded as primary protocols. After the behavioral test, the tested rats were then sacrificed, and the brain tissues were obtained for further biochemical tests. The water-maze tests for the APPswe mice were performed similarly, except the water pool used was 120 cm in its diameter and the water inside was made opaque by using dry milk.

### Western blotting

The animals were decapitated after the spatial memory retention test. The hippocampi were rapidly removed and homogenized at 4 °C using a Teflon glass homogenizer in 50 mM Tris-HCl, pH 7.4, 150 mM NaCl, 10 mM NaF, 1 mM Na_3_VO_4_, 5 mM EDTA, 2 mM benzamidine, 1 mM phenylmethylsulfonyl fluoride. The extract was mixed with sample buffer (3:1, v/v) containing 200 mM Tris-HCl, pH 7.6, 8% SDS, 40% glycerol, 40 mM DTT, boiled for 10 min, and then centrifuged at 12,000 × g for 10 min at 25 °C. The supernatant was stored at −80 °C for western blotting analysis. The protein concentration in the supernatant was estimated using a BCA kit according to the manufacturer’s instructions. The proteins were separated by 10% sodium dodecyl sulfate polyacrylamide gel electrophoresis (SDS-PAGE) and transferred to nitrocellulose membranes. The membranes were blocked with 5% nonfat milk dissolved in TBS-Tween-20 (50 mM Tris-HCl, pH 7.6, 150 mM NaCl, 0.2% Tween-20) for 1 h and probed with primary antibody at 4 °C for overnight. Then, the blots were incubated with anti-mouse or anti-rabbit IgG conjugated to horseradish peroxidase (1:5000) for 1 h at 37 °C and visualized with enhanced chemiluminescence. The blots were quantitatively analyzed using the Kodak Digital Science 1D software (Eastman Kodak Co., New Haven, CT, USA).

### Immunohistochemistry

For immunohistochemical studies, the rats were sacrificed using an overdose of chloral hydrate (1 g/kg) and perfused through the aorta with 100 ml 0.9% NaCl followed by 400 ml phosphate buffer containing 4% paraformaldehyde. The brains were removed and post-fixed in perfusate for 24 h. The rat brain sections embedded in paraffin were prepared for immunostaining through xylene treatment and gradual rehydration with 100–70% ethanol. Sections were blocked and then incubated with primary antibody overnight at 4 °C in 0.3% Triton X-100 phosphate buffered saline (PBS). The secondary antibody was incubated for 1 h at room temperature in the dark. The immunoreaction was detected using horseradish peroxidase-labeled antibodies for 1 h at 37 °C and visualized with the diaminobenzidine tetrachloride system (a brownish yellow color). For each primary antibody, 3–5 consecutive sections from each brain were used. The images were observed using a microscope (Olympus BX60, Tokyo, Japan).

### Golgi staining

Rats were perfused with 0.5% sodium nitrite solution and 4% formaldehyde. Brains were removed and stored in the dark for 2 days at RT in the same fixative, followed by storage in 1% silver nitrate solution for 3 days at room temperature. The brains were cut at 35 μm in the indicated orientation on a Minotome Plus microtome (Triangle Biomedical Sciences), dehydrated through 50%, 75%, 95%, and 100% alcohol, cleared in xylene, and then coverslipped.

### Nissl staining

The sections were stained in 1% toluidine blue solution for 20–40 minutes in a 50–60 °C oven, followed by differentiation in 95% ethyl alcohol for 10 minutes and dehydration in 100% alcohol. They were then cleared in xylene and coverslipped.

### Assay of AchE and ChAT activities

The activities of AchE and ChAT were determined by using rat AchE and rat ChAT ELISA kits according to the manufacturer’s instructions. The concentrations were interpolated from kit-specific standard curves generated using GraphPad Prism soft-ware (GraphPad Software, Inc., San Diego, California, USA).

### Assay of Aβ levels

The concentrations of Aβ42 and Aβ40 were determined using Mouse Aβ42 and Aβ40 ELISA kits by following the manufacturer’s instruction. The ratio of Aβ42/40 was determined by dividing Aβ40 by Aβ42 in the same sample.

### Preparation of synaptosomes

The hippocampi were homogenized in P2 buffer (10 mM Tris pH 7.4, 320 mM sucrose, 1 mM EDTA, 1 mM EGTA, 0.025% NaN3, 50 mm NaF, 2 mm Na3VO4), followed by centrifugation at 800 g for 5 min at 4 °C. The supernatants were centrifuged at 20,000 g for 15 min at 4 ºC. The sediment was used as the synaptosome fraction (P2).

### Imaging and statistical analysis

Image pro-plus software was used to calculate the number of Golgi stained dendritic spines and the immunohistochemical optical density. The data are expressed as the mean ± SEM and analyzed with SPSS 13.0 statistical software (SPSS Inc., Chicago, Illinois, USA). One-way ANOVA, followed by Student-Newman Keuls (SNK), was used to determine the statistical significance of differences among the means for multiple-group comparisons.

## Additional Information

**How to cite this article**: Wu, Y. *et al.* Intraperitoneal Administration of a Novel TAT-BDNF Peptide Ameliorates Cognitive Impairments via Modulating Multiple Pathways in Two Alzheimer's Rodent Models. *Sci. Rep.* **5**, 15032; doi: 10.1038/srep15032 (2015).

## Supplementary Material

Supplementary Information

## Figures and Tables

**Table 1:** The primary antibodies used for western blot analysis and immunohistochemistry.

Antibody	Specificity	Type	Dilution	Source
p-TrkB	Phosphorylated TrkB at Tyr706	pAb	1:1000	Santa Cruz
TrkB	Total TrkB	pAb	1:1000	Biovision
p-Erk1/2	Phosphorylated Erk1/2	pAb	1:1000	Cell Signaling
Erk1/2	Total Erk1/2	pAb	1:1000	Cell Signaling
p-Akt(473)	Phosphorylated Akt at Ser473	pAb	1:1000	Cell Signaling
Akt	Total Akt	pAb	1:1000	Cell Signaling
p-PKC	Phosphorylated PKC	pAb	1:1000	Cell Signaling
PKC	Total PKC	pAb	1:1000	Cell Signaling
p-CREB	Phosphorylated CREB at Ser133	pAb	1:1000	Cell Signaling
CREB	Total CREB	pAb	1:1000	Cell Signaling
Arc	Total Arc	pAb	1:1000	Calbiochem
pS396	Phosphorylated tau at Ser396	pAb	1:1000	Biosource
pS404	Phosphorylated tau at Ser404	pAb	1:1000	Biosource
pS262	Phosphorylated tau at Ser262	pAb	1:1000	Biosource
pT231	Phosphorylated tau at Thr231	pAb	1:1000	Biosource
pT205	Phosphorylated tau at Thr205	pAb	1:1000	Biosource
tau1	Nonphosphorylated tau	mAb	1:1000	Millipore
PP2A	Total PP2A	mAb	1:1000	Cell Signaling
p-PP2A(Y307)	Phosphorylated PP2A at Y307	mAb	1:1000	Abcam
t-APP	Total APP	mAb	1:1000	Millipore
p-APP	Phosphorylated APP at Thr668	pAb	1:1000	Cell Signaling
α/β-CTF	Anti-α/β-CTF	pAb	1:1000	Iqbal
PS1	Presenilin-1	pAb	1:1000	Cell Signaling
BACE1	BACE1	pAb	1:1000	Cell Signaling
DM1A	α-tubullin	mAb	1:2000	Sigma
4G8	Anti-Aβ	mAb	1:200	Iqbal
GSK-3	Total GSK-3α/b	pAb	1:1000	Cell Signaling
p-GSK3β(S9)	Phosphorylated GSK3β at Ser9	pAb	1:1000	Cell Signaling
p-GSK3β(Y216)	Phosphorylated GSK3β at Tyr216	mAb	1:1000	Upstate
P35/25	Total P35/25	pAb	1:1000	Cell Signaling
CDK5	Total CDK5	mAb	1:1000	Abcam
PSD93	Total postsynaptic density 93	pAb	1:1000	Abcam
PSD95	Total postsynaptic density 95	pAb	1:1000	Abcam
GluR1	Total GluR1	mAb	1:1000	Millipore
GluR2	Total GluR2	mAb	1:1000	Millipore
NR1	Total NMDA receptor 1	pAb	1:1000	Abcam
NR2A	Total NMDA receptor 2A	pAb	1:1000	Abcam
NR2B	Total NMDA receptor 2B	pAb	1:1000	Abcam
Synapsin I	Total Synapsin I	pAb	1:500	Millipore
Synaptophysin	Total Synaptophysin	mAb	1:1000	Sigma
M receptor	Total M1 receptor	pAb	1:100	Sigma
