# Supplementary material for: Intraperitoneal Administration of a Novel TAT-BDNF Peptide Ameliorates Cognitive Impairments via Modulating Multiple Pathways in Two Alzheimer’s Rodent Models
Source: Sci Rep. 2015 Oct 14;5:15032. doi: 10.1038/srep15032 (PMC4604491; doi:10.1038/srep15032)
Supplement: Supplementary Information [file srep15032-s1.pdf]

Supplementary information

Intraperitoneal Administration of a Novel TAT-BDNF Peptide Ameliorates Cognitive Impairments via Modulating Multiple Pathways in Two Alzheimer's Rodent Models

Yuanyuan Wu[a, b, 1], Xiaobin Luo[d, 1], Xinhua Liu[a], Deyi Liu[a], Xiong Wang[a], Ziyuan Guo[a], Lingqiang Zhu[a], Qing Tian[a], Xifei Yang[c,*], Jian-Zhi Wang[a, e,*]

[a]Department of Pathophysiology, School of Basic Medicine and the Collaborative Innovation Center for Brain Science, Key Laboratory of Ministry of Education of China for Neurological Disorders, Tongji Medical College, Huazhong University of Science and Technology, Wuhan 430030, China; [b]Department of Anesthesiology, Wuhan Children Hospital, Wuhan 430030 China; [c]Key Laboratory of Modern Toxicology of Shenzhen, Shenzhen Center for Disease Control and Prevention, Shenzhen, 518055, China; [d]Shenzhen/Guangzhou Kai-Tuo Biotech, Guangzhou, 510800, China; [e]Co-innovation Center of Neuroregeneration, Nantong 226001, China

[1]These authors contributed equally to this work.

[*]Correspondence to Professor Jianzhi Wang, Department of Pathophysiology, School of Basic Medicine and the Collaborative Innovation Center for Brain Science, Key Laboratory of Ministry of Education of China for Neurological Disorders, Tongji Medical College, Huazhong University of Science and Technology, Wuhan 430030, China, Tel: +86-27-83692625; Fax: +86-27-83693883; Email: wangjz@mails.tjmu.edu.cn; or Dr. Xifei Yang, Key Laboratory of

Modern Toxicology of Shenzhen, Shenzhen Center for Disease Control and Prevention, No. 8, Longyuan Road, Nanshan District, Shenzhen, 518055, China; Tel: +86-755-25601914, Fax: +86-755-25508584; Email: xifeiyang@yahoo.com.

Supplementary Fig. 1 | A diagram demonstrating the structure of the constructed fusion peptide.

Supplementary Fig. 2 | TAT-eGFP can efficiently penetrate the blood-brain barrier and was distributed in neural cells after intraperitoneal injection. (A) Images showing the distribution of eGFP that co-localized with the nuclei in hippocampal CA3, entorhinal cortex, hypothalamus, and amygdala of the rat brains. Blue: nuclei staining by Hoechst; Green: eGFP-labeled TAT. (B) Quantification of eGFP-positive cells in the entorhinal cortex, hippocampal CA1, CA2, and CA3 sub-regions, hypothalamus, and amygdala.

Supplementary Fig. 3 | The TAT-BDNF fusion peptide and galantamine effectively reversed the scopolamine-induced decline in the expression of synaptophysin. (A) Immunohistochemical images showing the distribution and expression of synaptophysin in the hippocampus (n=3). (B) Quantitative immunohistochemical analysis of the expression of synaptophysin. **$p<0.01$ *vs* Con group, ##$p<0.01$ *vs* Sco group.

Supplementary Fig. 4 | The TAT-BDNF fusion peptide reduced Aβ levels in APPswe mice. (A) Immunohistochemical analysis of the expression of Aβ in the mouse brain. Immunohistochemical images showing the expression of Aβ in the hippocampal regions CA1 and CA3 and cerebral cortex (n=3). (B) Quantitative immunohistochemical analysis. **$p<0.01$ *vs* APPwt group, ##$p<0.01$ *vs* APPswe group.

Supplementary Fig. 5 | Brain infusion of scopolamine and intraperitoneal injection of the TAT-BDNF fusion peptide do not cause cell death. No significant neuronal death in hippocampal CA1 and CA3, and DG, and cerebral cortex was observed among the Con, Sco, Pep, and Gal groups by Nissl staining (*n*=3).

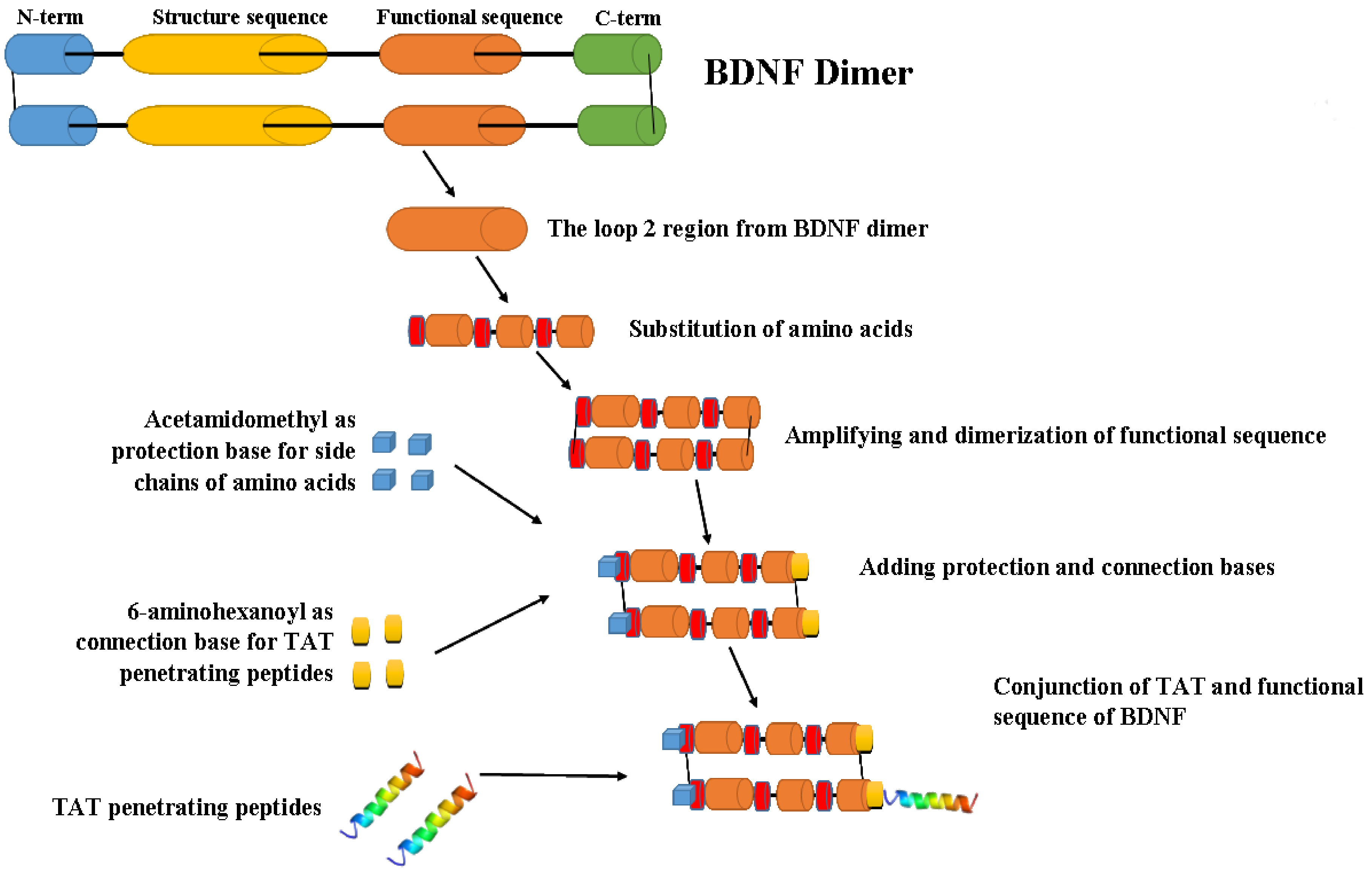

Supplementary Figure 1

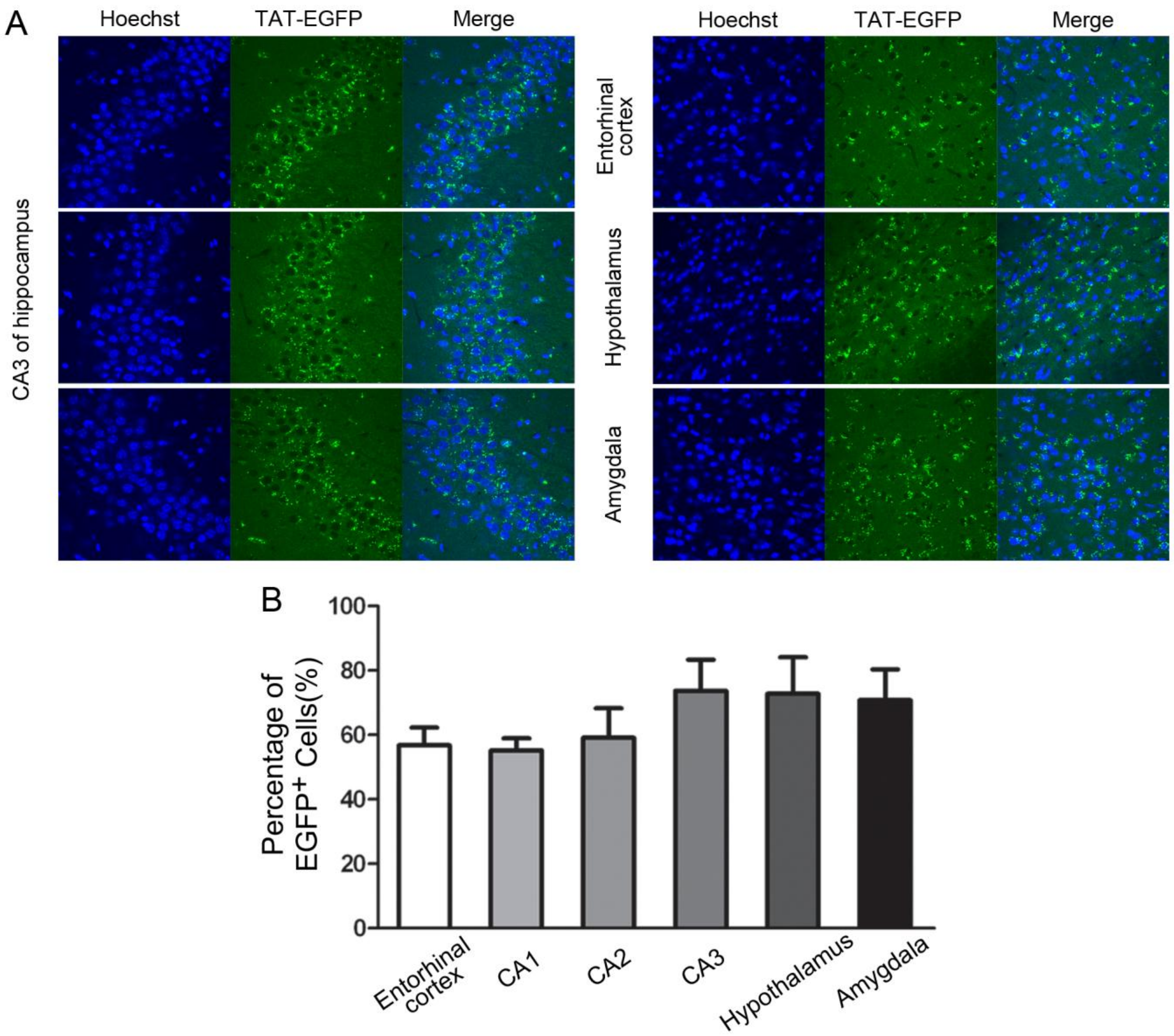

Supplementary Figure 2

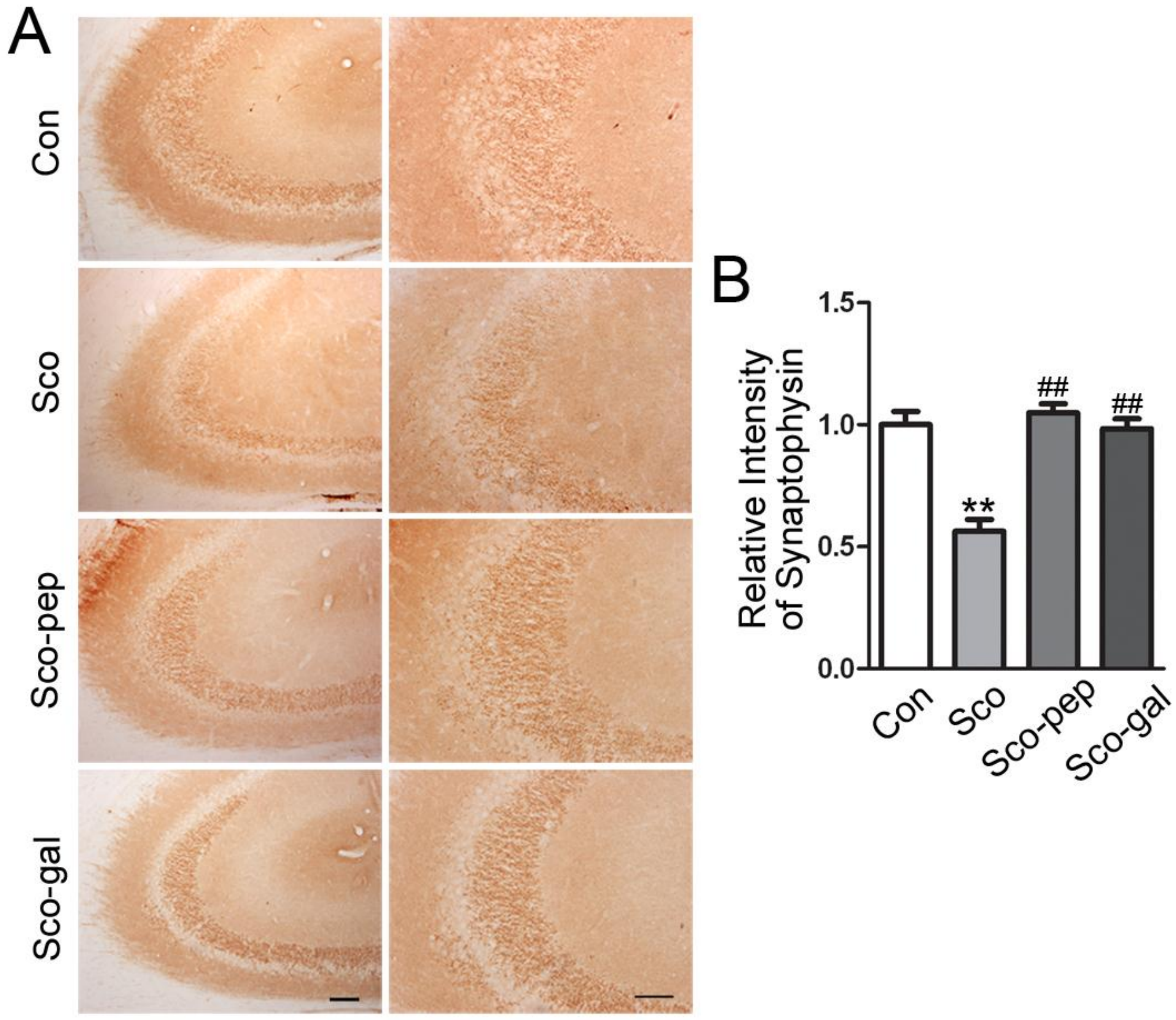

Supplementary Figure 3

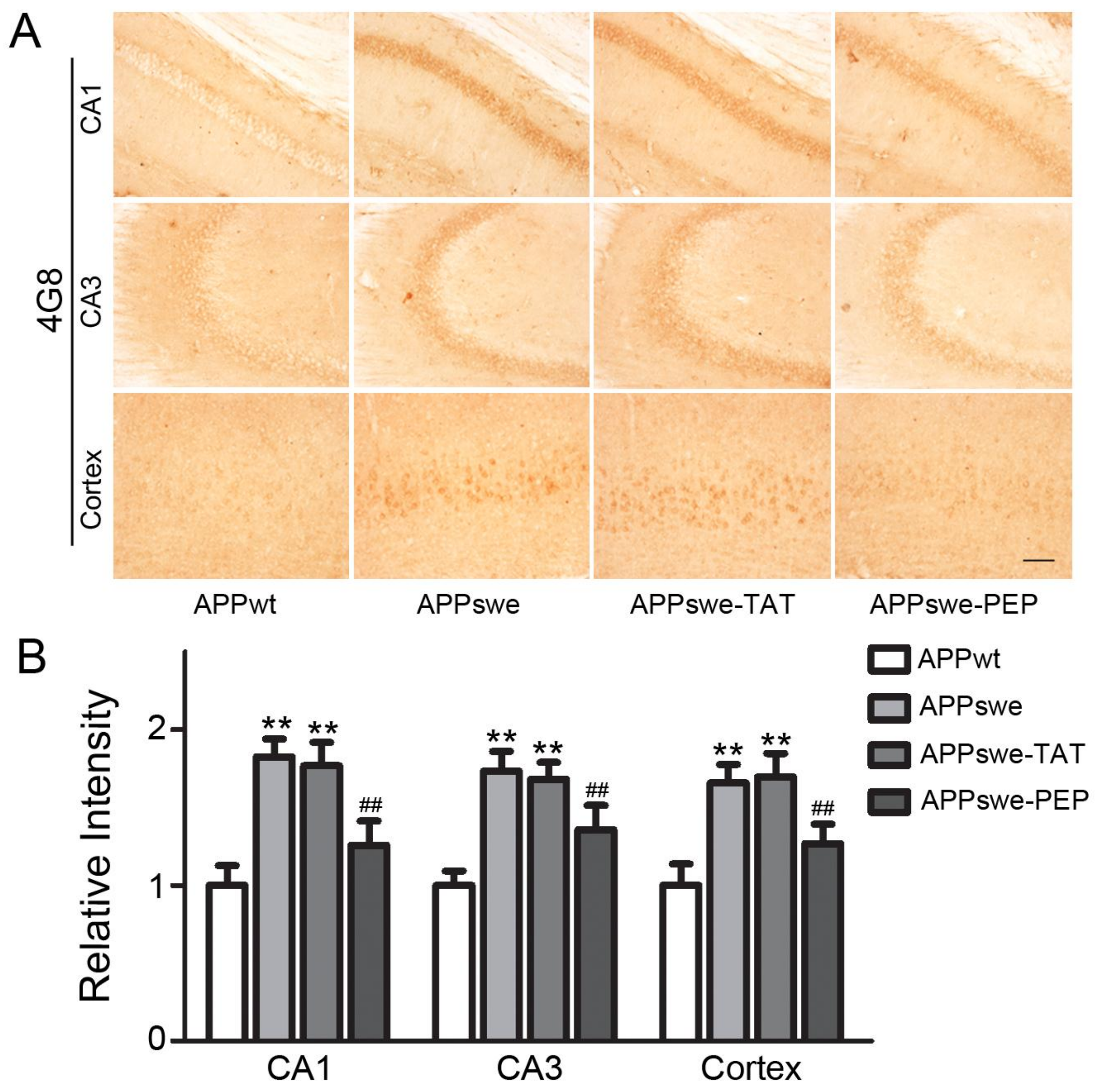

Supplementary Figure 4

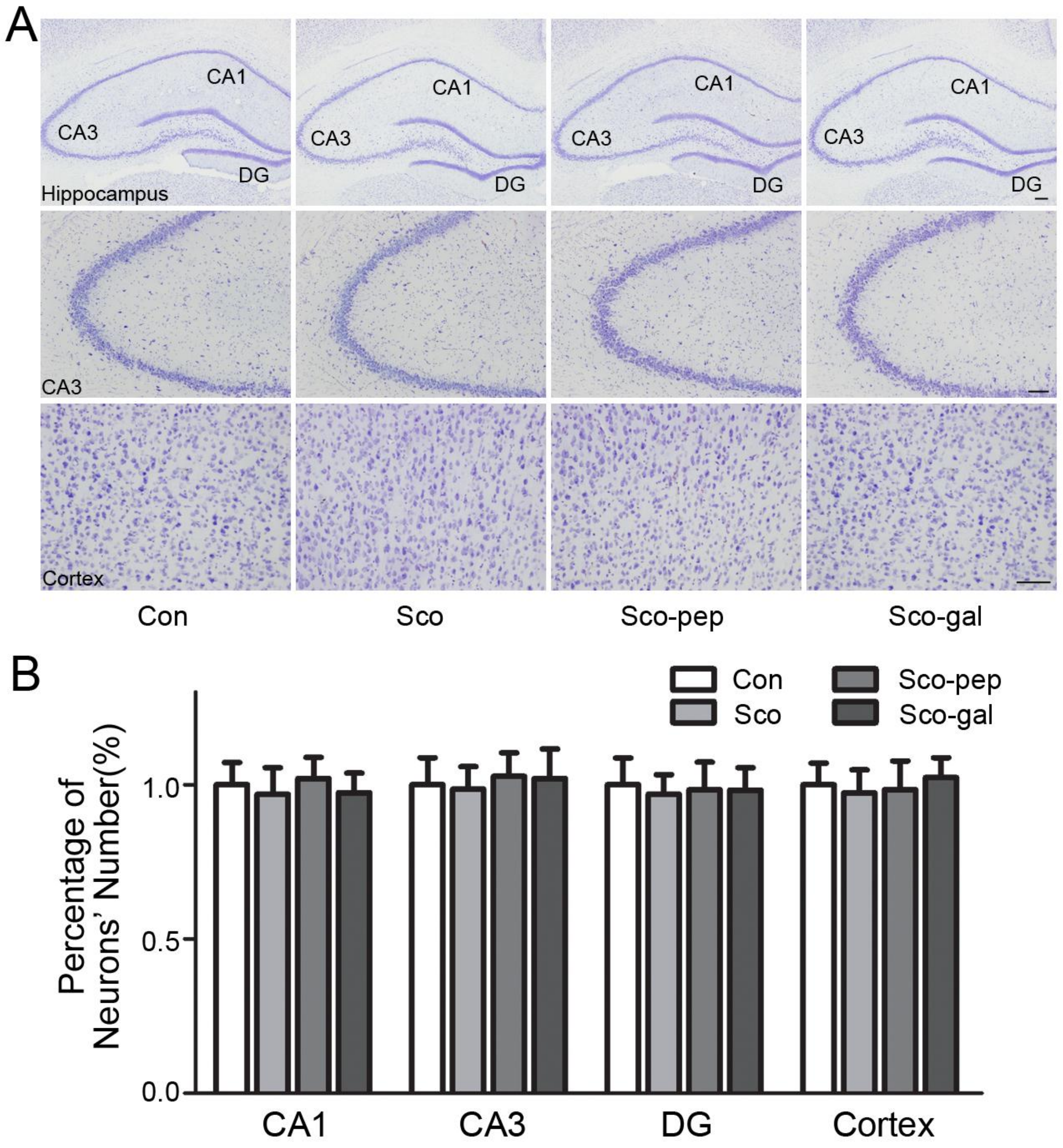

Supplementary Figure 5
